# The Effect of Mechanical Stress on Hyaluronan Fragments’ Inflammatory Cascade: Clinical Implications

**DOI:** 10.3390/life13122277

**Published:** 2023-11-29

**Authors:** Antonio Stecco, Lorenza Bonaldi, Chiara Giulia Fontanella, Carla Stecco, Carmelo Pirri

**Affiliations:** 1 Department of Physical Medicine and Rehabilitation, New York University School of Medicine, New York, NY 10016, USA; antonio.stecco@nyulangone.org; 2 Department of Civil, Environmental and Architectural Engineering, University of Padova, 35131 Padova, Italy; lorenza.bonaldi@phd.unipd.it; 3 Department of Industrial Engineering, University of Padova, 35121 Padova, Italy; chiaragiulia.fontanella@unipd.it; 4 Department of Neurosciences, Institute of Human Anatomy, University of Padua, 35121 Padova, Italy; carla.stecco@unipd.it

**Keywords:** mechanical stress, hyaluronan, hyaluronan fragment, inflammatory reaction, soreness, densification, fascial manipulation

## Abstract

It is a common experience, reported by patients who have undergone manual therapy that uses deep friction, to perceive soreness in treatment areas; however, it is still not clear what causes it and if it is therapeutically useful or a simple side effect. The purpose of this narrative review is to determine whether manual and physical therapies can catalyze an inflammatory process driven by HA fragments. The literature supports the hypothesis that mechanical stress can depolymerize into small pieces at low molecular weight and have a high inflammatory capacity. Many of these pieces are then further degraded into small oligosaccharides. Recently, it has been demonstrated that oligosaccharides are able to stop this inflammatory process. These data support the hypothesis that manual therapy that uses deep friction could metabolize self-aggregated HA chains responsible for increasing loose connective tissue viscosity, catalyzing a local HA fragment cascade that will generate soreness but, at the same time, facilitate the reconstitution of the physiological loose connective tissue properties. This information can help to explain the meaning of the inflammatory process as well as the requirement for it for the long-lasting resolution of these alterations.

## 1. Introduction

It is a common experience, reported by patients who have undergone manual therapy, to perceive soreness in treatment areas that lasts for around 24 h, with a numeric rating scale of < 3/10 [1]. However, this reaction cannot merely be considered as a side effect of treatment since it is supposed to be the expression of a fundamental inflammatory phenomenon that permits physiological tissue restoration [2]. One hypothesis is that the mechanical stresses of manual therapy, through deep friction, catalyze an inflammatory reaction that is key to restoring the proper viscosity of loose connective tissues with benefits in the physiology and functionality of the areas previously densified. In fact, from established nomenclature, the term densification defines an area perceived as rigid, rough, not sliding properly, and incompressible due to a viscosity alteration in the loose connective tissue typically localized between interfaces such as muscle and deep fascia or fascial system layers [3]. One method that supports the reorganization of the extracellular matrix and that has been demonstrated to be effective for densification treatment, thanks to deep friction manipulation, is the fascial manipulation (FM) method.

Whilst the treatment modality of the FM method can be compared to other techniques, the reasoning process for the choice of point to be treated presents major differences. The points are selected after a specific assessment process involving clinical history taking, clinical examination of specific movements, and, not least, palpatory verifications [4,5]. During the examination of clinical history, segments with dysfunctions are identified with an emphasis on chronology in order to generate a treatment hypothesis based on the current symptomatology of the patient and previous musculoskeletal events, which may be causing compensations. The selection of points to treat is guided by a specific assessment chart (FM chart) [5]. The choice of points of where to apply the deep friction is based on the information collected through the FM chart, movement, and, overall, palpatory verifications to define the presence of “densification”. These consecutive steps should limit the overall clinician’s subjectivity in the decision process [6]. The treatment must be performed over specific areas, called the center of coordination (CC) and the center of fusion (CF) which are anatomically safe and do not overlap major superficial nerves and veins. Additional guidance for point selection includes avoiding the patient’s excessively painful areas where inflammation, lesions, or even fractures could be present. Absolute contraindications are thrombosis, phlebitis, skin lesions, and fever [3]. The manipulation of the CC and CF has the aim of restoring the gliding of the underlying tissue layers [7].

As proof of this concept, a recent study demonstrated how FM can change the quality of the extracellular matrix in subjects with lateral elbow pain [4]. Specifically, this study showed how FM could decrease the free water concentrations proportionally to a patient’s symptoms (such as soreness). Physiologically speaking, attention has been paid to the altered composition of the extracellular matrix in painful conditions due to hyaluronan (HA) status which saturates its water-bound links due to a self-aggregation tendency.

HA, historically regarded as a mere “space-filler” within the extracellular matrix (ECM), has undergone a remarkable transformation in our understanding of its significance. Emerging research has unveiled its profound structural and signaling roles [8]. This seemingly unassuming molecule is universally distributed among vertebrates and plays a pivotal role, especially within the ECM of soft connective tissues [9]. HA, the simplest glycosaminoglycan (GAG), is composed of a non-sulfated linear polymer, consisting of as many as 20,000 repeats of its disaccharide unit, which combines D-glucuronic acid and N-acetyl-D-glucosamine [10]. Thanks to its carboxyl groups, HA carries a negative charge and exhibits remarkable hydrophilicity, enabling it to retain water molecules at an astonishing 1000-fold of its own molecular weight [10]. This newfound understanding opens doors to exciting avenues of research and applications in various fields. HA is a dynamic molecule with a turnover rate that varies across different tissues, ranging from 12 to 24 h in skin tissues to mere minutes within the bloodstream [11]. This dynamic turnover is pivotal in regulating the molecular weight of HA, which, in turn, influences its properties and functions. HA’s equilibrium between synthesis and degradation is a crucial factor in maintaining tissue homeostasis.

At high molecular weights (HMWs), HA forms a substantial, viscous network. When it interacts with various proteoglycans, such as aggrecan, it leads to the creation of molecular composites that occupy significant volumes within the ECM. These complexes contribute to the gel-like state of the matrix. Additionally, these extensive HA–HA binding proteoglycan complexes also crosslink with other matrix proteins, including collagen. This crosslinking results in the formation of supermolecular structures that significantly enhance tissue stiffness [12]. The intricate interplay between HA, proteoglycans, and collagen within the ECM has profound implications for tissue structure and function. HA’s HMW and its ability to form these complex supermolecular structures make it a critical regulator of tissue rigidity. Moreover, the dynamic turnover of HA in different tissues underscores its versatility and adaptability, allowing it to fulfill specific roles tailored to the needs of each tissue type [12]. Understanding the intricate biology of HA at the molecular level provides a foundation for exploring its potential therapeutic applications and enhancing our knowledge of tissue physiology and ECM dynamics. Research in this field continues to uncover the multifaceted roles of HA and its impact on tissue health, regeneration, and overall well-being.

The ECM undergoes significant alterations in its physical properties, particularly concerning the presence of free water and the entangled HA chains. This leads to a substantial increase in viscosity within the ECM, which, in turn, has profound implications for the behavior of loose connective tissue and the mechanisms governing interactions between adjacent tissue interfaces [13]. It is crucial to recognize that the viscosity of HA is inherently temperature dependent. As the temperature surpasses the threshold of 40 °C, the three-dimensional superstructure of HA chains progressively disintegrates [13]. This disintegration results in a subsequent reduction in viscosity. Conversely, at lower temperatures, the viscosity of HA increases. This temperature-driven modulation of viscosity can have clinical ramifications, spanning from the onset of simple muscle stiffness and a decreased range of motion to more complex consequences such as the irritation of free nerve endings and the generation of painful afferent signals [14].

Understanding the temperature-induced alterations in HA viscosity provides valuable insights into the physiological responses of connective tissues and the dynamic nature of the ECM. These insights are particularly pertinent in clinical contexts, where interventions and treatments are designed to mitigate the adverse effects of temperature-induced changes [14]. By delving into the intricacies of HA behavior in response to temperature fluctuations, researchers and medical professionals can devise targeted strategies to address continuously involved connective tissue, pain management, and mobility impairment more effectively. Furthermore, this knowledge fuels investigations into the development of therapeutic approaches that leverage the tunable properties of HA to optimize patient outcomes and overall tissue health. As research in this domain continues to evolve, the potential applications of understanding HA viscosity dynamics extend into diverse medical and biomechanical fields, promising innovative solutions for various health challenges [13,14].

For instance, Menon et al. (2019) demonstrated the direct correlation between water-bound HA and range of motion in spastic patients [15]. These authors injected human recombinant hyaluronidase, which has the capacity to fragmentize the long chains of HA, to drain and metabolize the exceeded amount of self-aggregate HA with poor hydrophilic capacity. As a result, a more homogenous fluid was formed, stimulating the local cells to produce new HA with regular water-bound capacity and allowing proper sliding between interfaces [15].

Similar restoration of range of motion has also been observed in different studies applying manual therapy in musculoskeletal pain patients [3,4,5,6]. Following these clinical results, it was postulated that even manual therapy, such as FM, should be able to catalyze the above degradation of the long-entangled chain of HA. The friction generated is postulated to generate mechanical stress that could fragmentize HA, catalyzing the same process as hyaluronidase [15].

**Figure 1:**
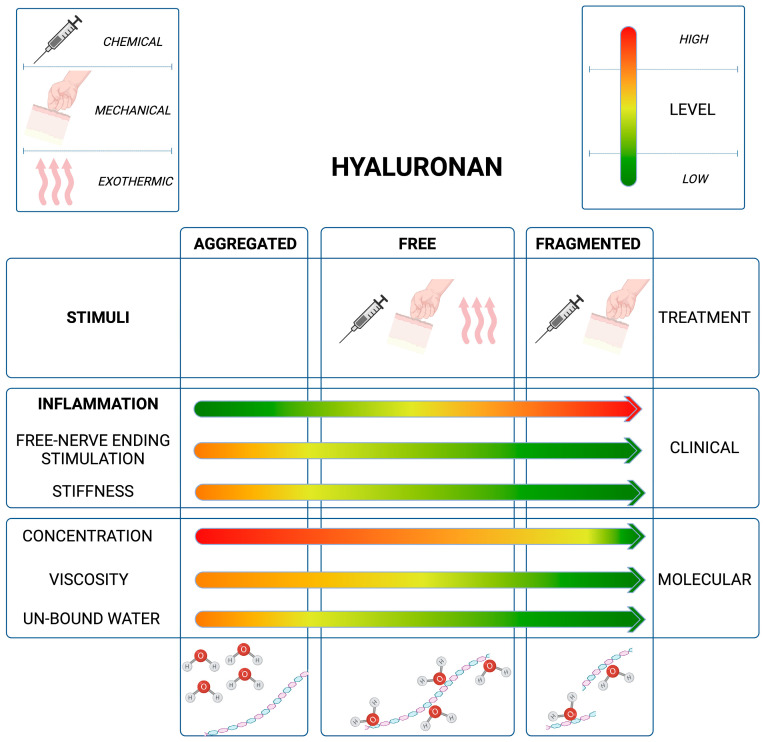
Graphical representation of patients’ symptom relief proportional to the decrement in HA self-aggregation tendency and the subsequent reduction in the amount of free (unbounded) water, triggered by mechanical stimuli and in accordance with HA fragmentation. (Image created using software BioRender, agreement number: PO263KPOYR).

The full reversible process, which takes approximately a few days (less than one week), transforms a painful and stiff area into a softer region for the therapist and a less painful or unpainful region for the patient. Indeed, Cowman et al. [7] have also suggested that patients, who have undergone manual treatment, will still perceive soreness for up to 24–48 h even if the drop in pain and stiffness occurs in the first few minutes (Figure 1).

The purpose of this article is to define if mechanical stress can catalyze an inflammatory process, driven by HA fragments, with soreness as a clinical manifestation. Specifically, the reaction should metabolize self-aggregate HA chains, responsible for loose connective tissue viscosity increasing, not only catalyzing a local HA fragment cascade that will generate soreness but also reconstituting the physiological loose connective tissue properties.

## 2. Materials and Methods

**Figure 2:**
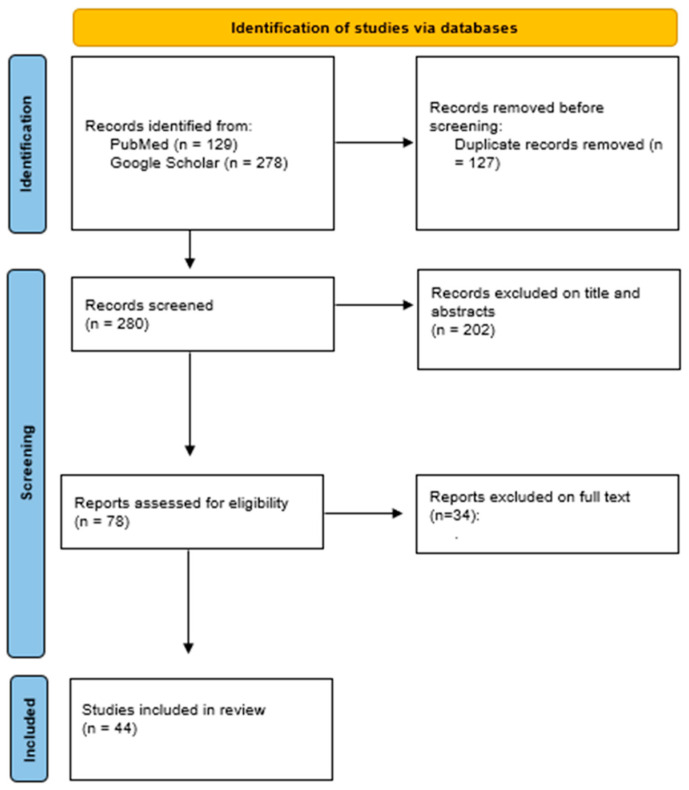
PRISMA flow diagram for study selection.

A substantial literature search was conducted to review the literature from 1960 through March 2023. The studies included in this review were identified by searching on PubMed and Google Scholar (n = 44) using the following words: “hyaluronan fragments” AND/OR “hyaluronic inflammation” AND/OR “hyaluronan oligosaccharides” AND “Mechanical stresses” AND “manual therapy” AND “physical therapy”. All relevant English publications without any category restrictions were included. Relevant secondary references were also captured. The papers were evaluated according to their title, abstracts, and text (n = 407), with duplicates being removed (n = 278). Finally, 44 papers were considered that deal with hyaluronan inflammation in different extra-articular anatomical structures. The results were reported in Figure 2 according to new PRISMA statements. In detail, the three reviewers (A.S., L.B., and C.P.) involved in the research of the articles carried out their activity blind to each other, also considering hyaluronan inflammation, biomechanical issues, or mechanical stress caused by manual therapies and physical therapy.

## 3. Results

### 3.1. The Role of HA Weight: A Decremental Cascade during Inflammation

HA, in normal constitution, provides viscoelasticity and lubrication of liquid connective tissues [16]. Because of these properties, HA is able to lubricate and space-fill tissues [17] with a fundamental role for the constitutional ECM organization [18] present within endomysium, perimysium, epimysium interfaces, and deep fascia layers. The variability of HA concentration in the human body can range from less than 40 ng/mL in blood serum [19] to about 2–3 mg/mL in the knee synovial joint [20]. HA synthesis is driven by different enzymes [21]; one of them is hyaluronan synthase 2 which has been reported to be able to generate HA as large as 6000 kDa, the typical average size for newly synthesized HA in healthy tissues [22]. Indeed, the HA between 1800–3000 kDa, also known as high-molecular-weight hyaluronan (HMW HA), is responsible for tissue hydration due to its ability to bind high amounts of water. Nonetheless, HA can retain not only water up to almost 1000 times its weight but also self-aggregate and bind many proteins [23,24,25]. When ECM homeostasis is altered, endogenous HMW HA is disrupted, unbalancing the equilibrium toward a higher concentration of medium-molecular-weight HA (MMW HA, 250–1000 kDa) to low-molecular-weight HA (LMW HA, ≤250 kDa). Then, LMW HA can be further fragmented into shorter oligomers (o-HA, <10 kDa) [26]. A variety of different authors have demonstrated how mechanical stresses can be the root cause of HA depolymerization from high to low molecular weight [27]. Other authors [28] have also proven that HA chains can be broken by physical shearing or physical stress (high-speed stirring or critical shearing) [29]. Nazet et al. [30] have shown HA reduction under advanced stretching conditions due to reduced displacement or increased hyaluronidase activity.

### 3.2. The Inflammation Cascade: Influencing Factors

HA can also undergo depolymerization via non-specific mechanisms. Inflammatory processes can lead to the generation of free radicals within tissues undergoing widespread inflammation [31]. When the natural antioxidant defenses prove insufficient to counter the substantial influx of ROS, these radicals directly interact with native HA, resulting in significant production of HA fragments [32].

In a proposed catabolic pathway [33], it is suggested that the high-molecular-mass HA polymer undergoes stepwise cleavage by a series of enzymes, with the product of one reaction becoming the substrate for the subsequent one. These successive enzymatic events result in the generation of increasingly smaller HA fragments. Small HA components are able to exacerbate the inflammatory response by inducing the release of various detrimental mediators such as ROS, cytokines, chemokines, and destructive enzymes (i.e., hyaluronidase) and by facilitating the recruitment of leukocytes [34]. HA fragments, with a molecular size ≤ 500 kDa, have been shown to exhibit several proinflammatory effects. They can stimulate the expression of proinflammatory genes including TNFa, IL-1β, IL-1, and MMPs [35]. LMW HA can further maintain and strengthen the inflammatory response [34]. In particular, the presence of LMW HA stimulates the production of proinflammatory cytokines [36]. As proof of this concept, it has also been proven that HA fragments, in the range of 200–250 kDa, can induce the expression of inflammatory cytokines [33]. Yamamoto et al. [37] described a new cell surface hyaluronidase (hyal), acting at pH 6–7, that degrades HMW HA into an intermediate size (~5 kDa) promoting the inflammatory cascade. Therefore, the induction of such inflammatory mediators by HA, enhances the pre-existing inflammatory response, creating a positive feedback loop where inflammation promotes further inflammation [38].

Yamasaki et al. [39] also showed that during sterile inflammatory processes, HA is able to activate the interleukin 1β (IL-1β) pathway and the cryopyrin mechanism. Previous reports have shown that HA fragments can stimulate an inflammatory response through their interaction with the TLR-4 and CD44 receptors [34]. The same authors explain how HA fragments, produced from native HA degradation, mediate a response made by IL-1β that produces an inflammatory response through the CD44 receptor [34]. The activation of these receptors mediated the activation of the nuclear factor kB (NF-kB) which in turn activates the release of several proinflammatory cytokines. HA fragments are also generated by hyal2, which is present on the cell membrane together with CD44. [40]. Hyal2 translocation is required for the degradation of extracellular hyaluronan. In a quiescent state, the hyal2 resides predominantly in intracellular space. However, activation of the CD44 signaling pathway results in the translocation of hyal2 to the cellular membrane, enabling the degradation of extracellular HA [41]. Hyal2 enzymatically cleaves high-molecular-weight HA, reducing it to a final product of approximately 20 kDa, which corresponds to approximately 50 disaccharide units [33]. The hyaluronan fragments generated by hyal2 are then internalized, transported to endosomes, and ultimately delivered to lysosomes, where hyal1 further breaks down the 20-kDa fragments into smaller disaccharides [33]. β-exoglycosidases actively contribute to the degradation of 20-kDa HA fragments throughout the entire catabolic cascade, rather than solely at the final stages [33].

### 3.3. HA Polymer Fragments: Diverse Biological Activities

While commonly categorized as proinflammatory, it is more appropriate to consider HA fragments as pro-defensive entities in specific environments [9]. HA polymer fragments exhibit diverse biological activities depending on their size and are integral to numerous essential processes [33]. Studies have shown that short oligosaccharides often play a role in the body’s alarm system [42]. Moreover, some of the smaller HA oligosaccharides appear to alleviate the effects of these stress signals [33]. For instance, six-unit oligosaccharides derived from HA exhibit the capacity to stimulate fibroblast motility and expedite wound closure [43]. In contrast, HMW HA (1500 kDa) inhibits platelet adhesion and the activation of endothelial cell layers. HA oligomers also possess the ability to Impede the proliferation and migration of vascular smooth muscle cells in response to platelet-derived growth factor, as elucidated by Tavianatou et al. [38]. Additionally, oligomeric Ha fragments prompt the production of various cytokines by both macrophages and fibroblasts, thereby fostering cellular migration and facilitating the process of wound healing [44]. Numerous other researchers have corroborated that the anti-inflammatory properties of LMW HA can mitigate the inflammatory response, while Tavianatou et al. [38] have substantiated the claim that oligomeric HA reduces HA accumulation. These HA fragments can effectively bind to CD44 and consequently attenuate a multitude of signaling pathways that lead to diverse biological responses [45,46]. Misra et al. [47] demonstrated that o-HA can significantly reduce the activation of several receptor tyrosine kinases. Kolar et al. [48] unveiled that combinations of degraded HA fragments effectively inhibit specific inflammatory responses in both cellular and murine models. Remarkably, the smallest HA oligosaccharides demonstrate an inhibitory effect on inflammatory response elicited by lipopolysaccharides (LPS), resulting in a reduction in proinflammatory cytokines and an elevation in anti-inflammatory cytokines. It is imperative to note that the HA fragments do not invariably yield pro-inflammatory effects or another signaling effect, as they may also trigger defensive cellular responses mediated by Toll-like receptors (TLRs) [19]. Xu et al. [49] ascertained that exceedingly minute HA oligosaccharides, induce the expression of heat shock proteins and possess anti-apoptotic properties, effectively suppressing cellular apoptosis. Furthermore, the medical administration of the hyaluronidase enzyme does not incite an inflammatory response [50].

**Figure 3:**
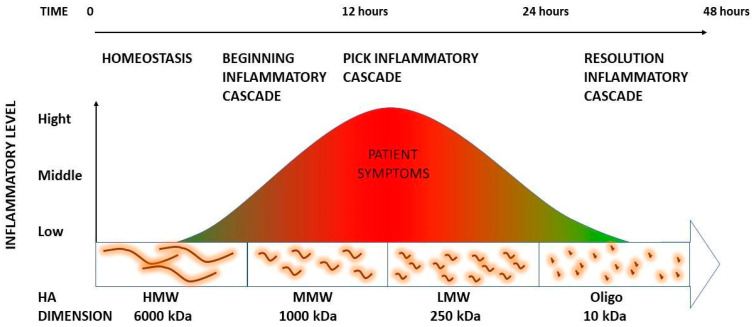
Schematization of hyaluronan fragments and corresponding inflammation.

All of these studies help to explain how the inflammatory cascade, when catalyzed by manual therapy or free radicals, is then able to self-resolve (Figure 3). To finalize the process, HA pieces will then either be further depolymerized locally or drained from the tissue via the lymphatic system [34]. Most of the HA fragments leave the tissue with the lymph and are cleared in the lymph nodes. All that remains, after passage through the nodes, is degraded by the liver [51].

## 4. Discussion

Manual therapies and physical therapies are the most common therapeutic options for non-specific musculoskeletal pain even if a great variety of treatment options are available. Furthermore, the duration of the results is often questionable due to relative short-term effects [52]. While McDevitt et al. [52] in their recent review proved the efficacy of physical therapies within 6 weeks, only a few therapies have demonstrated long-term effects [6,53].

Matteini et al. [54] showed how HA aggregation breaks down progressively when the temperature increases over ~40 °C. These values are compatible with weak non-covalent interactions like those characteristics of van der Waals and hydrophobic forces localized between oligo HA fragments. This information can easily explain how low energy therapies (exothermic or light manual therapies) can decrease HA aggregation so the entire viscosity of the extracellular matrix as a consequence improves the range of motion and decreases the irritation of the free nerve ending [54]. However, the long chains of HA cannot be washed out without being fragmented first. This could explain the recurrence of symptoms due to consequent re-aggregation of the HA that will still be retained in the compartment due to the lack of mechanical forces that will stimulate the drainage and consequently metabolization of the fragments.

On the other hand, high-intensity therapy such as focus shock wave and deep friction manipulation can not only warm up the area, separating the single HA chains and giving an instantaneous result, but also fragmentize the HA. The latter catalyzes an inflammatory process that metabolizes entangled HA chains that are more susceptible to absorbing physical forces due to their overstructured conformation.

Huin-Amargier et al. [28] and Capila I. and Sasisekharan R. [29] proved that HA chains can be broken by physical shearing stress. This supports the capacity of FM or, more so in general, manual therapies to catalyze the specific inflammatory processes that are needed to substitute the incorrect HA with new ones made by the fasciacytes [55].

In a nutshell, in the course of inflammation, HA undergoes depolymerization, breaking down into smaller fragments with LMW. A significant portion of these fragments subsequently undergo further degradation, resulting in the formation of small oligosaccharides. These oligosaccharides possess the capacity to exacerbate the inflammatory process by stimulating the generation of various inflammation mediators and agents, including ROS, cytokines, chemokines, and destructive enzymes (such as hyaluronidase). These enzymes serve to initiate and intensify the inflammatory response [56].

HMW HA species are prominent in the earliest stages, whereas more fragmented forms are generated progressively until the end of the process [38]. The LMW HA, which exacerbates the inflammatory process, explains the 12 h soreness peak that the patients will perceive. This also explains how inflammation does not originate from any anatomical lesions but rather from the manual treatment consequence of ECM. The smallest products of the HA catabolic cascade can turn about and suppress the action of larger predecessors, mollifying their effects [57]. The hypothesis, which should be experimentally proven, is that it can resolve the inflammatory process that was previously catalyzed by manual treatment and exacerbated by LMW HA. It was proven that the metabolic pathways for HA synthesis and degradation are highly ordered. They are composed of carefully controlled reactions that rely on the regulation of individual enzyme activities [57]. For this reason, we recommend considering the above inflammatory reaction as a “physiological” phenomenon that has a high therapeutic value for maintaining manual treatment results. For instance, FM treatments were able to demonstrate statistically significant differences at 3, 6, and even 9 months follow-up [6,58,59]. These results, defined as the range of motion increasing and symptoms decreasing, were reached thanks to better extracellular sliding and a consequent decrease in free nerve ending irritation. High-intensity therapy should be able to generate not only a viscosity decrease, through HA chain separation, but also fragmentation of the incorrect, exceeded amount of HA that can then be drained and metabolized in the lymphatic system. Quick HA turnover, with 33% daily replacement [60] facilitated by the fasciacytes [55], can then restore the correct HA amount and conformation in each treated area. Nevertheless, the mechanical normalization of the interface space, between fascial layers, can also facilitate fasciacyte stimulation which represents a key element in the healing process.

## 5. Conclusions

This narrative review was able to support a common outcome of manual therapies that use deep friction like FM. The soreness that patients perceive in the next 24 h seems to correlate with the HA cascade that will restore the normal physiology in the treated area. This information can help to explain the meaning of the inflammatory process as well as its requirements. We can hypothesize that the inflammatory cascade should be a critical component of the healing process in all of these patients who present densifications. We can suppose that the more chronic the densification is, the more HA aggregation will be present and, consequently, the longer the manipulation required to resolve it will be [61], as shown in how the time used to restore normal gliding can vary from some seconds to a few minutes. While manipulation of a couple of minutes should only catalyze an inflammatory process without manifesting its intensity during treatment, longer manipulation, past 5 min, can easily generate local inflammation that can be perceived immediately by the patient. Furthermore, the higher the inflammatory process that is generated, the longer the patient’s soreness will be perceived. Clinical practice has shown wide variability in soreness duration with a minimum of 24 h up to 72 h but occasionally even longer times of soreness duration due to a variety of other factors [1]. Indeed, we can assume that the duration of inflammation can also be defined by other factors like the efficiency of the patient’s immune system and the activities performed after the treatment as well as the patient’s pain perception. A weak immune system, such as in patients who present fever without clear infections, can eradicate the resolution of the reaction. On the opposite, some warm-up activities, such as sports activities or stretching, can accelerate the inflammatory cascade. Being aware of this process becomes fundamental for both therapists, who are now justified to generate it, and patients who should be informed of its benefits. The inflammatory process should not be confused with erroneous treatment strategies that may exacerbate symptoms in neighboring body segments. Furthermore, this inflammatory cascade should not be stopped with non-steroid anti-inflammatory drugs, if chronic therapies have not yet been established, but the opposite is facilitated. Ideally, non-steroid anti-inflammatory drugs should be substituted with paracetamol which still has an analgesic effect but should not stop the specific inflammatory process presented in this manuscript. Further studies should be conducted to strengthen this hypothesis.
